# Overexpression of serine acetyltransferase in maize leaves increases seed‐specific methionine‐rich zeins

**DOI:** 10.1111/pbi.12851

**Published:** 2017-11-29

**Authors:** Xiaoli Xiang, Yongrui Wu, José Planta, Joachim Messing, Thomas Leustek

**Affiliations:** ^1^ Department of Plant Biology Rutgers University New Brunswick NJ USA; ^2^ Institute of Biotechnology and Nuclear Technology Sichuan Academy of Agricultural Sciences Chengdu China; ^3^ National Key Laboratory of Plant Molecular Genetics CAS Center for Excellence in Molecular Plant Sciences Institute of Plant Physiology & Ecology Shanghai Institutes for Biological Sciences Chinese Academy of Sciences Shanghai China; ^4^ Waksman Institute of Microbiology Rutgers University Piscataway NJ USA

**Keywords:** serine acetyltransferase, seed storage protein, transgenic, zein, methionine, nutritional quality

## Abstract

Maize kernels do not contain enough of the essential sulphur‐amino acid methionine (Met) to serve as a complete diet for animals, even though maize has the genetic capacity to store Met in kernels. Prior studies indicated that the availability of the sulphur (S)‐amino acids may limit their incorporation into seed storage proteins. Serine acetyltransferase (SAT) is a key control point for S‐assimilation leading to Cys and Met biosynthesis, and SAT overexpression is known to enhance S‐assimilation without negative impact on plant growth. Therefore, we overexpressed *Arabidopsis thaliana AtSAT1* in maize under control of the leaf bundle sheath cell‐specific *rbcS1* promoter to determine the impact on seed storage protein expression. The transgenic events exhibited up to 12‐fold higher SAT activity without negative impact on growth. S‐assimilation was increased in the leaves of SAT overexpressing plants, followed by higher levels of storage protein mRNA and storage proteins, particularly the 10‐kDa δ‐zein, during endosperm development. This zein is known to impact the level of Met stored in kernels. The elite event with the highest expression of *AtSAT1* showed 1.40‐fold increase in kernel Met. When fed to chickens, transgenic *AtSAT1* kernels significantly increased growth rate compared with the parent maize line. The result demonstrates the efficacy of increasing maize nutritional value by SAT overexpression without apparent yield loss. Maternal overexpression of SAT in vegetative tissues was necessary for high‐Met zein accumulation. Moreover, SAT overcomes the shortage of S‐amino acids that limits the expression and accumulation of high‐Met zeins during kernel development.

## Introduction

Maize (*Zea mays L*.) is a primary grain commodity with 1053.8 million metric tons projected worldwide production in 2016/2017 (USAD/FAS report January 2017, Grain: World Markets and Trade). By 2050, maize production is expected to increase by 10%, whereas per capita consumption is expected to increase by 63% (http://maize.org/why-maize/). A key shortcoming of maize as a food source is that it is deficient in the essential amino acids such as lysine (Lys), tryptophan (Trp) and methionine (Met). For this reason, formulation of maize‐based livestock feed includes the addition of soya bean, which complements Trp and Lys deficiency. However, soya bean does not supply sufficient Met, which is added to feed as a racaemic mixture. The current total worldwide Met market is 685–700 KMT and is expected to increase by 27% in 2018 (http://www.feedinfo.com/files/novus-white-paper.pdf). Therefore, a means to increase the Met content of maize would be a significant benefit for the animal feed market. Nutritionally enhanced maize would also benefit subsistence farmers in developing countries who rely on maize as a staple food.

The amino acid content of maize kernels is mainly determined by the expression of a set of seed storage proteins termed zeins that are produced during grain development and are deposited in endosperm into structures, termed protein bodies. Zeins belong to the superfamily of prolamins and have evolved into four classes based on their chemicophysical properties, termed alpha (α), beta (β), gamma (γ) and delta (δ) zeins (Xu and Messing, 2009). The α‐zein genes have many copies in the genome that can vary in numbers between different inbred lines, whereas the others range between one and two copies (Dong *et al*., 2016). The amino acid composition of the specific zeins differs markedly. Most zeins have very few Met and cysteine (Cys) residues. Only five zeins have higher numbers of Cys and Met residues. The 50‐kDa, 27‐kDa and 16‐kDa γ‐zeins contain 6.48%, 7.84% and 9.20% Met + Cys residues, respectively, the 15‐kDa β‐zein contains 15.63% Met+Cys, and the 18‐kDa and 10‐kDa δ‐zeins contain 26.84% and 26.3% Met + Cys (Wu *et al*., 2012). Even though these zeins determine the Met + Cys content of maize kernels, they normally make up only a small proportion of the total complement of seed storage proteins. The level of Met in kernels varies significantly among different inbred lines ranging from sufficient to insufficient levels in animal feed. This genetic variation is controlled by quantitative trait loci (QTLs). However, none of the QTLs have yet been cloned (Deng *et al*., 2017).

Maize zeins are also essentially devoid of two other essential amino acids, Lys and Trp. The *opaque2* and *floury2* mutant seeds have a higher content of these amino acids resulting from elevated expression of nonzein proteins, combined with reduced expression of the most abundantly expressed α‐zeins (Mertz *et al*., 1964). Reduced α‐zeins render kernels soft, making them unfit for commercial production. Hard endosperm of *opaque2* mutant lines has been restored by breeders, which is referred to as quality protein maize (QPM) that has higher Lys and Trp (Vasal *et al*., 1980). But QPM maize is still Met‐deficient (Cromwell *et al*., 1967).

One potential method of manipulating the S‐amino acid composition of maize kernels is to engineer overexpression of Met‐rich seed storage proteins. Overexpression of the 10‐kDa δ‐zein significantly increased the Met content, but at the expense of β‐ and γ‐zein expression and reduced Cys content (Lai and Messing, 2002; Wu *et al*., 2012), indicating that the availability of S‐amino acids limits the total accumulation of the S‐amino acids in zeins. A similar phenomenon of S‐amino acid reallocation resulting from a limitation of S‐amino acids was described by others who have attempted to overexpress S‐rich storage proteins in crop plants (Chiaise *et al*., 2004; Hagan *et al*., 2003).

Another potential method of increasing S‐amino acid content of maize kernels is to reduce the expression of specific seed storage proteins. When β‐ and γ‐zein expression is silenced through RNAi, the 10‐kDa δ‐zein accumulates to a higher level, resulting in a significant increase in the total seed Met content (Wu and Messing, 2010; Wu *et al*., 2012). These results illustrate that it is possible to shift the amino acid content of kernels by altering expression of individual zeins that change the allocation of S‐amino acids. Nevertheless, S‐amino acid supply remains a limitation to production of zeins with high Met + Cys content.

Cys and Met are the end products of the sulphur (S) assimilation pathway 0 (Takahashi *et al*., 2011). In brief, S‐assimilation occurs when sulphide reacts with O‐acetyl‐L‐serine (OAS) to form Cys, catalysed by OAS‐thiol‐lyase (OASTL). Met is formed from Cys in three additional steps. The primary biosynthetic control point for Cys includes sulphate reduction by APS reductase (APR) and OAS formation by serine acetyltransferase (SAT). Met synthesis is controlled by cystathionine‐γ‐synthase in some, but not all species (Haas *et al*., 2008; Kim *et al*., 2002; Leustek and Saito, 1999; Leustek *et al*., 2000; Tabe *et al*., 2010). Transgenic *A. thaliana* overexpressing APR accumulated intermediates of S‐assimilation including sulphite (SO_3_^2−^), sulphide (S^2−^), thiosulphate (S‐SO_3_^2−^), as well as the pathway end products Cys and glutathione (Tsakraklides *et al*., 2002). The same transgenic strategy in maize produced a marked increase in S‐assimilation, but at the expense of plant vigour, which was attributed to the accumulation of toxic S intermediates (Martin *et al*., 2005). Moreover, the accumulation of the S intermediates suggested that sulphate reduction outpaced OAS production in the APR overexpressing plants. However, these negative growth effects can be overcome by expressing E. coli APR in leaf cells (Planta et al., 2017).

Transgenic overexpression of SAT, necessary for OAS synthesis, has also been shown to increase S‐assimilation in a variety of crop plant species (Blaszczyk *et al*., 1999; Harms *et al*., 2000; Nguyen *et al*., 2012; Tabe *et al*., 2010), but without the negative effects on plant growth observed with APR overexpression (Martin *et al*., 2005; Tsakraklides *et al*., 2002). The stimulation of S‐assimilation was attributed to a twofold effect: (i) increased OAS providing the direct substrate for increased Cys synthesis, and (ii) OAS also functions as a positive regulator of expression of S‐reduction genes (Ohkama‐Ohtsu *et al*., 2004), resulting in increased S^2−^ necessary for reaction with OAS to form Cys.

Here, we describe the analysis of transgenic maize lines overexpressing SAT. In C4 plants such as maize, S‐assimilation is known to occur in bundle sheath cells (Kopriva and Koprivova, 2005). Therefore, SAT overproduction was engineered using the bundle sheath cell‐specific Rubisco small subunit (*rbcS1*) promoter (Sattarzadeh *et al*., 2010). The effect of overexpressing SAT in bundle sheath cells was to increase S‐assimilation without apparent negative impacts on plant growth. In addition, we found that this transgenic strategy resulted in the elevated accumulation of the 10‐kDa δ‐zein, as well as the 27‐kDa γ‐zein and 15‐kDa β‐zein proteins in both developing and mature seeds. In particular, the expression of the 10‐kDa δ‐zein is known to be a prime contributor to Met content of kernels (Wu and Messing, 2010; Wu *et al*., 2012). The net result was a significant increase in protein‐bound Met in mature kernels and an increase in the nutritional value measured in a chicken feeding trial. In total, overexpression of SAT in maize has the effect of, not only enhancing S‐assimilation, but also, indirectly impacting expression of high‐Met seed storage proteins.

## Results

### Transformation of maize with *AtSAT1*

**Figure 1:**
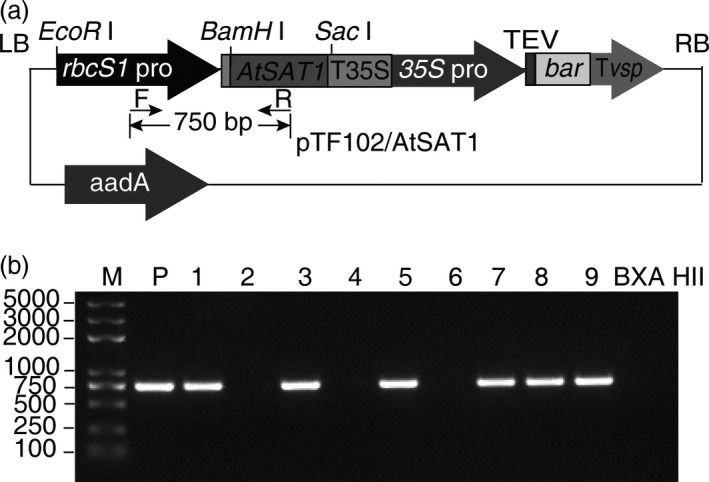
AtSAT1 transformation of maize. (a) Schematic diagram of the SAT overexpression construct. The construct components include the T‐DNA right border, RB; and left border, LB; the bundle sheath cell‐specific Rubisco small subunit 1 promoter, *rbcS1* promoter; the *A. thaliana* serine acetyltransferase1 coding sequence, *AtSAT1*; the CaMV 35S terminator, T35S; and the phosphinothricin acetyltransferase cassette consisting of the 35S promoter; tobacco etch virus translational enhancer, TEV; the bar gene; and the soya bean vegetative storage protein terminator, Tvsp. (b) PCR confirmation of T_1_ transformants. The DNA templates used for PCR amplification include the vector plasmid, or genomic DNA from each of nine transformants or the nontransformed line BXA HII, which is the maize line used for transformation.

To investigate overexpression of SAT in maize, the *A. thaliana SAT1* (*AtSAT1*) coding sequence was placed under the control of the *rbcS1* promoter, which drives expression specifically in bundle sheath cells (Sattarzadeh *et al*., 2010). Because the *AtSAT1* protein lacks a transit peptide, it should result in cytosolic accumulation of the protein. *AtSAT1* was chosen as the target of overexpression because of prior studies by the senior authors’ laboratory with the *A. thaliana* enzyme (Murillo *et al*., 1995; Sors *et al*., 2005). The rbcS1p::*AtSAT1* expression cassette was cloned into binary vector pTF102 (Frame *et al*., 2002) (Figure 1a), and this plasmid was used to transform immature embryos of F_1_ progenies of the cross between the maize HiII lines of B and A (HiII Parent B × Parent A F_1_).

### Identification of *AtSAT1* expression lines

The primary phosphinothricin‐resistant transgenic plants from independent transformation events (derived from different immature embryos) were tested for the presence of the transgene by PCR amplification using genomic DNA as template. Six of the nine transgenic events that were tested contained the *AtSAT1* transgene (Figure 1b). Those plants were grown and a fully expanded leaf used for the measurement of SAT activity and SAT protein by immunoblotting. All lines showed significantly higher SAT protein level as measured by immunoblotting than the parent maize line (Figure S1a), and in addition, all of the transgenic plants showed significantly higher SAT activity (Figure S1b).

**Figure 2:**
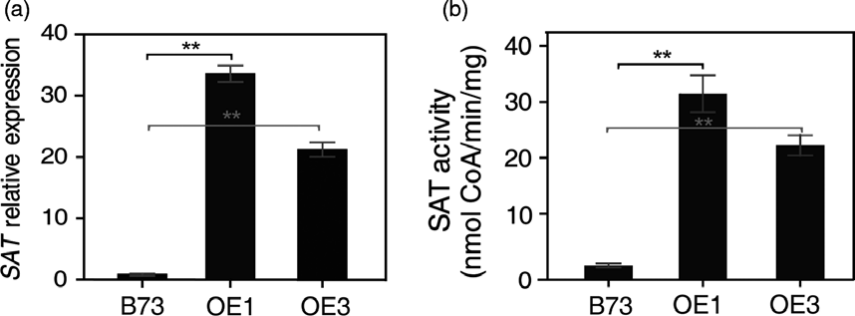
Maize plant expression of *AtSAT1*. (a) Quantitative RT‐PCR of two independent transgenic lines OE1 and OE3. Both OE1 and OE3 for this and all subsequent experiments, unless noted otherwise, were the result of two backcrosses to the maize inbred line B73 and then selection of nonsegregating plants. For this reason, B73 was used as the nontransgenic control. RNA was extracted from young leaves of two‐month‐old plants, and *AtSAT1* was amplified with specific primers. The *Actin* primers were used as reference gene control. (b) SAT activity in the leaves of OE1 and OE3. The data in graphs (a) and (b) represent the mean of three measurements from different plant samples±SD. The specific activity of crude extracts is given in nmol CoA produced per min and mg total protein. Asterisks indicate significant differences between B73 and transgenic plant lines using the one‐way ANOVA function of GraphPad Prim (*P *< 0.001).

Two lines, OE1 and OE3, derived from transgenic event #1 and #3 (Figure 1b), were selected for further analysis. These T_1_ plants were grown to maturity, backcrossed for two generations with maize inbred line B73, then self‐pollinated for selection of transgenic nonsegregating lines (T_3_ generation). Unless noted otherwise, the T_3_ nonsegregating plants were used for all subsequent analyses. Both OE1 and OE3 were used to measure *AtSAT1* mRNA level by qRT‐PCR, and the same plants were used to measure SAT enzyme activity. Figure 2 shows that the expression of *AtSAT1* mRNA (Figure 2a) and SAT enzyme activity (Figure 2b) was much higher in OE1 and OE3 lines than parental B73. In addition, the plants derived from OE1 showed higher *AtSAT1* mRNA and higher SAT enzyme activity than OE3. It should be noted that the measured enzyme activity is a combination of endogenous SAT and that derived from expression of *AtSAT1* (Figure 2b), whereas only *AtSAT1* mRNA was measured in Figure 2a. These results show that *AtSAT1* was stably expressed over multiple generations.

### Sulphur assimilation end products accumulate in leaves of *AtSAT1* transgenic maize

**Figure 3:**
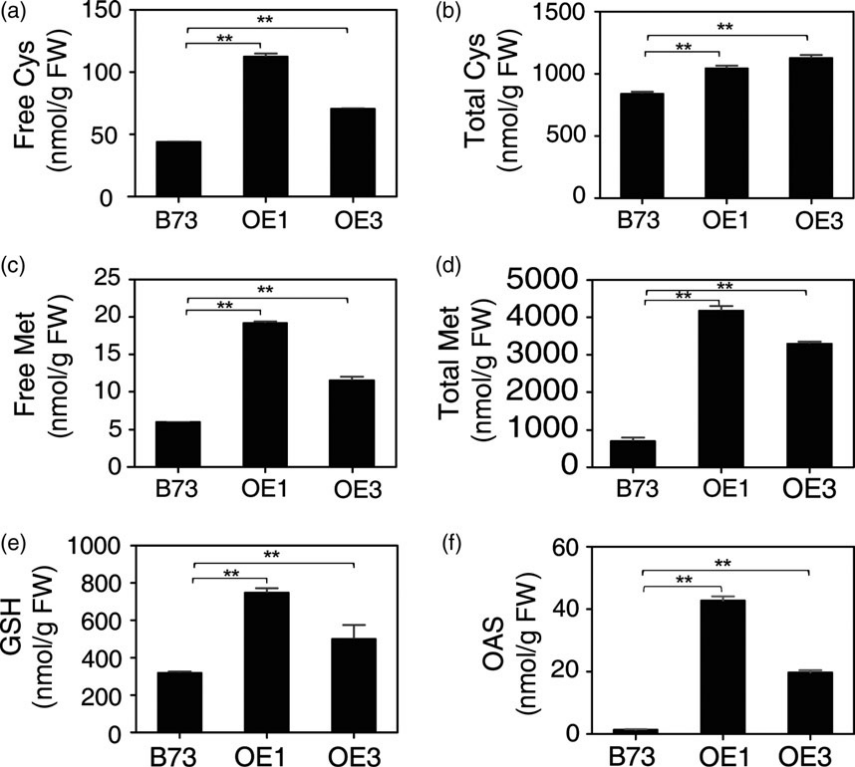
Sulphur metabolites in *AtSAT1* transgenic maize. Sulphur metabolites in leaves of 2‐month‐old transgenic *AtSAT1* lines OE1, OE3 and parental B73. (a) The level of free Cys. (b) The level of total Cys. (c) The level of free Met. (d) The level of total Met. (e) The level of total glutathione. (f) The level of OAS. The values are the mean of three independent biological replicates ±SD. Asterisks indicate significant differences from B73 (Student's *t*‐test, *P* < 0.05).

The leaves of 2‐month‐old OE1 and OE3 plants were examined for the contents of S metabolites. When compared with parental B73, free Cys and Met were found to be approximately twofold higher in OE1 and OE3 (Figure 3a and b). Total (free and protein‐bound) Cys was slightly, but significantly increased, and Met was up to fourfold higher (Figure 3c and d). Also, glutathione was found to be twofold to threefold higher (Figure 3e). These results show that overexpression of *AtSAT1* has resulted in an increase in S‐assimilation end products in the transgenic lines. That Met accumulates in OE1, and OE3 suggests that Met synthesis in maize is not strictly controlled by the enzyme cystathionine γ‐synthase (CGS). CGS is also not a limitation for Met synthesis in potato (Kreft *et al*., 2003), whereas it is a bottleneck in Arabidopsis (Chiba *et al*., 1999; Kim *et al*., 2002; Lee *et al*., 2005).

### Sulphate reduction is increased in leaves of *AtSAT1* maize

**Figure 4:**
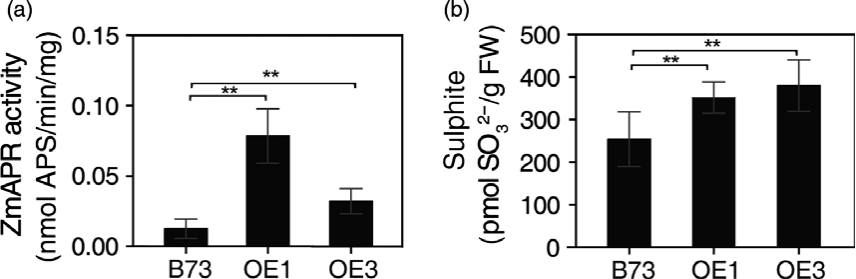
Sulphate reduction in *AtSAT1* transgenic maize. APR activity and sulphite in leaves of 2‐month‐old transgenic *AtSAT1* lines OE1, OE3 and parental B73. (a) *ZmAPR* enzyme activity. (b) Sulphite content. Samples were from T_1_ transgenic maize lines OE1 and OE3. The values are the mean of three independent biological replicates ±SD. Asterisks indicate significant differences from B73 (Student's *t*‐test, *P* < 0.05).

When SAT was overexpressed in potato and *A. thaliana,* a pleiotropic increase in APR was observed (Hopkins *et al*., 2005; Hubberten *et al*., 2012; Sirko *et al*., 2004). The increase in APR expression was attributed to elevated OAS, the product of the SAT enzyme, which, in addition to its role in Cys synthesis, also functions as a positive signal for expression of sulphate reduction genes. To assess whether SAT overexpression has the same effect in maize as in *A. thaliana* and potato, OAS and sulphite were measured as was the activity of APR. OAS was significantly higher in leaf of OE1 and OE3 compared with the parental B73 (Figure 3f). APR activity was also significantly higher (Figure 4a) as was the content of sulphite (Figure 4b). Sulphite is the product of APR and might be expected to accumulate given that APR activity was higher in the *AtSAT1* plants. Therefore, we conclude that SAT overexpression in maize has a similar effect as it does in other plant species (Hubberten *et al*., 2012).

### Mature kernels of *AtSAT1* overexpressing maize show changes in zein expression profile

**Figure 5:**
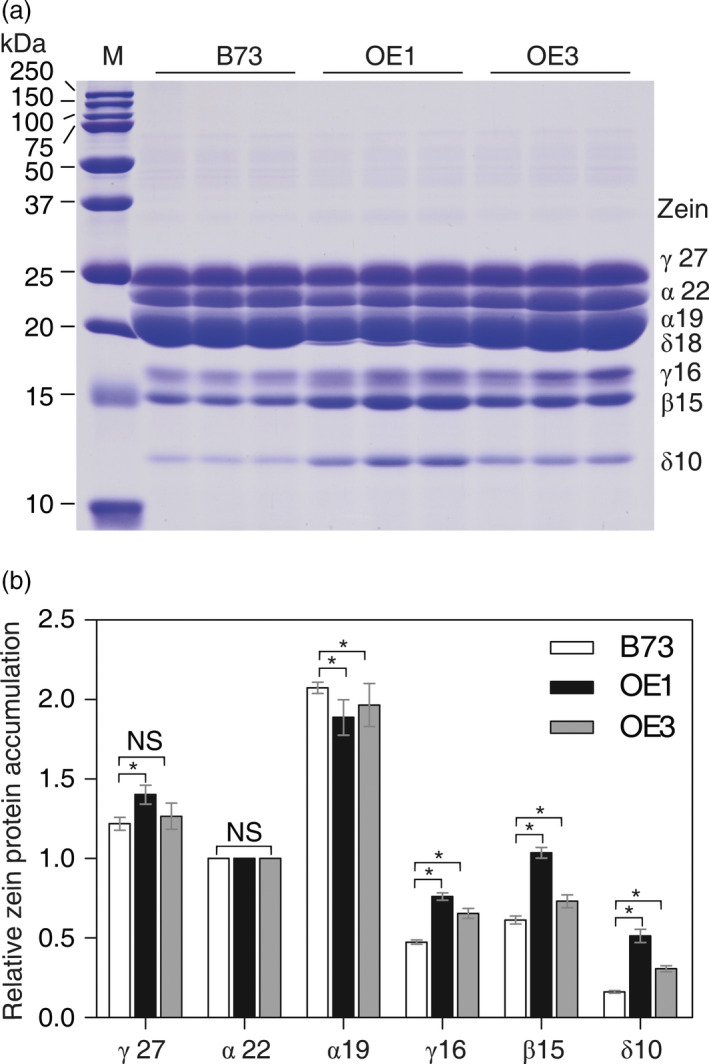
Zein accumulation in transgenic kernels. (a) Kernels from OE1 and OE3 were harvest from field plants. The kernels were fully mature, and protein profiles from three different kernels harvested from different plants are shown. Protein from 300 μg dry weight of endosperm sample was loaded in each lane. M, protein markers from top to bottom being 250, 150, 100, 75, 50, 37, 25, 20, 15 and 10 kDa. The mass of each zein is indicated to the right of the figure. (b) The relative abundance of zein proteins was analysed from SDS‐PAGE analysis from three different kernels harvested from different plants using the densitometry function of ImageJ software. The band intensities were normalized using the 22‐kDa zein and the results plotted as relative values ±SD. Asterisks indicate significant differences from B73 (Student's *t*‐test, *P* < 0.05).

S‐amino acid supply was thought to limit accumulation of high‐Met zeins and the storage of S‐amino acids in maize seed (Lai and Messing, 2002). Therefore, the seed storage protein profile of the seeds produced by the collection of T_1_ *AtSAT1*‐expressing primary transgenic maize was analysed by SDS‐PAGE. The seeds from all of the transgenic plants showed increased expression of the 10‐kDa δ‐zein (Figure S1c, d), whereas the other zeins were unaffected (although a slight increase in the 16‐kDa γ‐zein, 15‐kDa β‐zein was noted). The 10‐kDa δ‐zein has the highest content of Met + Cys residues (26.84%, Wu *et al*., 2012). In order to determine to whether the increase in the 10‐kDa δ‐zein in *AtSAT1* expressing maize is heritable, the zein profiles of seeds produced by nonsegregating T_3_ plants OE1 and OE3 were analysed. The profile of zeins is shown in Figure 5a, and the densitometric quantification of the protein bands is shown in Figure 5b. The results reveal that the 10‐kDa δ‐zein is markedly increased in both transgenic lines. The 15‐kDa β‐zein and 16‐kDa γ‐zein, two other zeins are also increased in both transgenic lines. Other changes in the zein profile included small, but reproducible increase in the 27‐kDa γ‐zein, and slight but reproducible decreases in the 19‐kDa α‐zein. The 22‐kDa α‐zein, one of the proteins with the lowest Met + Cys content, was unchanged. The effect of *AtSAT1* expression on the zein profile change appears to be specific to the high Met + Cys class of proteins because low Met + Cys zeins expression was unaffected as was the nonzein protein profile of endosperm and embryos (Figure S2).

### *AtSAT1*‐associated changes in zein profile improve nutritional value

Given the increased expression of 10‐kDa, 15‐kDa and 16‐kDa zeins in *AtSAT1* kernels, it was of interest to determine whether kernel nutritional value is improved. The amino acid content of mature kernels represents the culmination of gene expression throughout seed development. To assess the effects of *AtSAT1* overexpression on maize kernel amino acid composition, the total amino acids content in hydrolysed kernel flour from OE1 and OE3 were compared with B73. Table 1 shows that the Met and Cys contents from both OE1 and OE3 were significantly increased. In OE1, Met was 1.40‐fold higher compared with kernels from B73 and Cys was 1.32‐fold higher. In OE3, Met was 1.20‐fold higher, and Cys was 1.32‐fold higher. The content of all other amino acids was found to be the same or very slightly reduced.

**Table 1:** Amino acid composition analysis of maize kernels

	AA_ab_(±SD)		
	B73	OE1	OE3
Methionine	0.25 (0.02)	0.35 (0.05)	0.30 (0.02)
Cysteine	0.19 (0.02)	0.25 (0.02)	0.25 (0.01)
Lysine	0.35 (0.02)	0.34 (0.02)	0.37 (0.02)
Phenylalanine	0.60 (0.02)	0.61 (0.05)	0.62 (0.07)
Leucine	1.41 (0.05)	1.40 (0.16)	1.42 (0.14)
Isoleucine	0.38 (0.02)	0.40 (0.06)	0.40 (0.05)
Threonine	0.47 (0.04)	0.45 (0.03)	0.40 (0.03)
Valine	0.62 (0.04)	0.60 (0.06)	0.67 (0.05)
Histidine	0.31 (0.03)	0.31 (0.04)	0.36 (0.04)
Arginine	0.52 (0.06)	0.43 (0.05)	0.45 (0.06)
Glycine	0.42 (0.04)	0.41 (0.05)	0.47 (0.03)
Aspartic Acid	0.76 (0.05)	0.75 (0.07)	0.88 (0.11)
Serine	0.62 (0.03)	0.60 (0.07)	0.67 (0.07)
Glutamic Acid	2.65 (0.19)	2.41 (0.58)	2.63 (0.60)
Proline	0.98 (0.05)	1.03 (0.13)	1.03 (0.02)
Alanine	0.94 (0.03)	0.88 (0.10)	0.96 (0.10)
Tyrosine	0.37 (0.02)	0.36 (0.05)	0.38 (0.03)
Total	11.84 (0.57)	11.58 (0.46)	12.26 (0.36)

Amino acid values are expressed as the percentage of total amino acids in the sample (AA_ab_) with standard deviations in parentheses. The values are the averages from three independent measurements ±SD in parentheses. Each assayed sample was from kernels taken from a single ear of nonsegregating OE1 and OE3 in B73 background.

**Figure 6:**
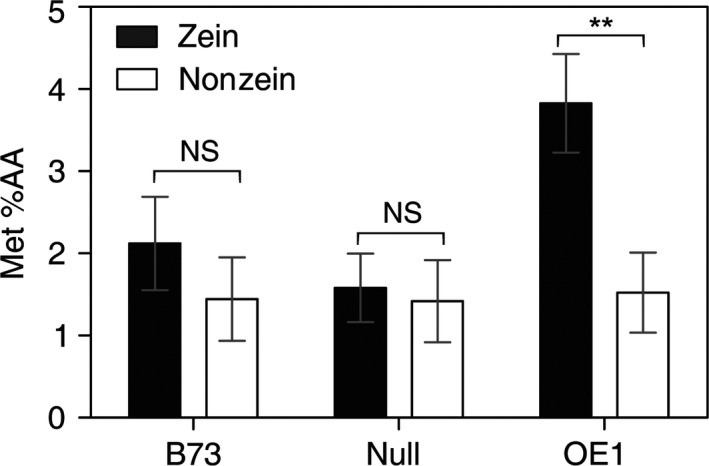
Met content measured with a bacterial biosensor. The Met content of maize zein and nonzein proteins was measured from B73 and plants derived from a segregating population of OE1. Each value is the mean of three individual plants ±SD. Asterisks indicate significant differences from B73 (Student's *t*‐test, *P* < 0.05).

To examine whether the increase in total Met correlates with an increase in bioavailable Met, a Met biosensor assay was used. The biosensor assay measures the bioavailable Met used for growth of a Met‐auxotrophic *Escherichia coli* strain, so it is a measure of the nutritional value of the kernel samples. Unfortunately, an equivalent *E. coli* Cys biosensor does not exist. The results in Figure 6 show that Met content is significantly increased specifically in the zein fraction derived from OE1 kernels compared to null sergeant or the B73 parent. By comparison, the bioavailable Met content of the nonzein fraction was nearly identical in B73 and OE1, indicating that the increase in Met is specific to the zein protein fraction and that the Met content of the zein fraction is in a bioavailable form.

**Figure 7:**
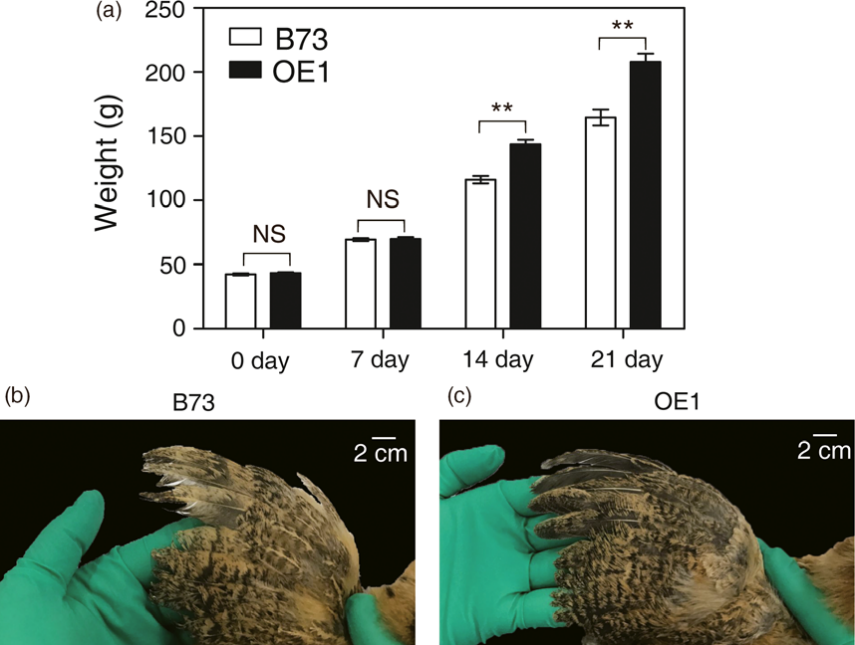
Chicken feeding trial with OE1. (a) Growth rate of chicks fed a diet formulated with OE1 kernels or kernels from B73 segregant plants. The data shown are the means from twenty‐one chicks ±SD. Asterisks indicate significant differences in OE1 compared with B73 (Student's *t*‐test, *P* < 0.01). (b,c) Close‐up image of 21‐day‐old primary wing feathers.

To further test the nutritional value of OE1 kernels, a chicken feeding trial was performed. The growth of chickens is a facile model for testing Met nutritional value of animal feed (Lai and Messing, 2002; Messing and Fisher, 1991). Two different maize flour‐based diets were formulated to feed Erlang Mountain chicks, one with flour from B73 kernels and the second with flour from OE1 kernels. Aside from the different plant sources of the maize flour, the formulated diets were prepared identically. The chicks were fed for up to 21 days and their weights periodically measured. Compared with the feed that was formulated with normal B73 kernels, chicks fed a diet formulated with OE1 kernels grew significantly faster as evidenced by weight measurements performed at 14 days and 21 days (Figure 7a and Table S1). Adequate dietary Met is required for robust feather growth. Indeed, diminished feather growth is an early symptom of dietary Met deficiency in chickens. Figure 7b, c shows that wing feathers are much longer from chicks fed the OE1‐kernel diet compared with the B73 kernel diet.

To achieve common use of this new high‐Met trait, it is necessary that the transgene does not negatively impact yield. Examination of the OE1 transgenic line revealed that there was no significant change compared with null segregants in respect to several parameters including average grain yield per plant, seed weight, ear length, plant height, number of rows per ear and kernels per row (Figure S3).

### Reciprocal crosses reveal that zein profile correlates with maternal *AtSAT1* expression

**Figure 8:**
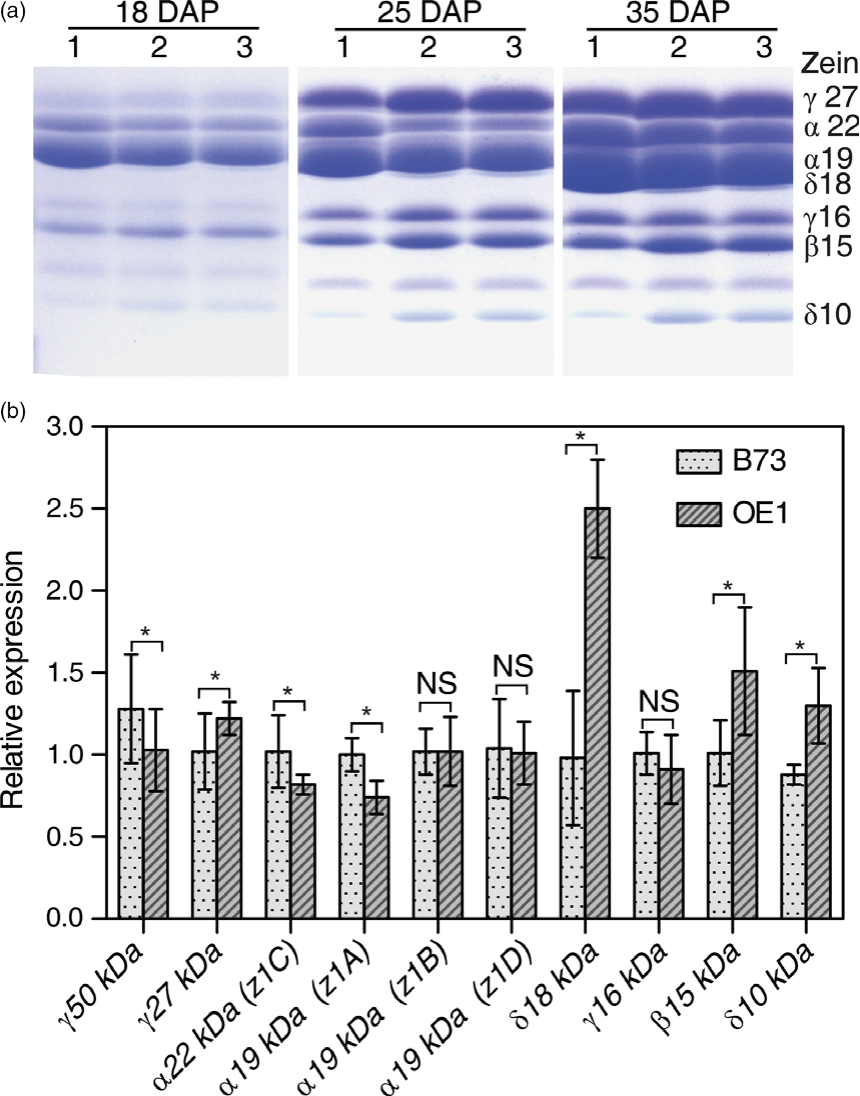
Maternal *AtSAT1* is necessary for accumulation of 10‐kDa δ‐, 15‐kDa β‐ and 18 kDa δ‐zeins. (a) SDS‐PAGE analysis of zein proteins in endosperm of B73xOE1, OE1xB73 and self‐pollinated OE1. The samples were harvested at 18 DAP, 25 DAP and 35 DAP. Endosperm was isolated, and zein was extracted. Total zein loaded in each lane was equal to 300 μg of maize fresh weight. (b) Zein mRNA was measured in endosperm sampled 18‐DAP by quantitative RT‐PCR. Relative expression of zein genes in OE1 compared to B73. The data are the mean from three independent plants per transgenic line ±SD. Asterisks indicate significant differences from B73 (Student's *t*‐test, *P* < 0.01).

Given that the increase in Met content and nutritional value of kernels from plants expressing AtSAT1 was found to result from changes in zein profile, it was of interest to define the constraints of the relationship between SAT and zeins. Self‐pollinated and reciprocal crosses of OE1 and parental B73 were studied to determine the inheritance of the change in zein profile associated with *AtSAT1* expression. Self‐pollinated OE1 was compared to crosses of parental line B73 and OE1 in which the transgenic plant was either the maternal (OE1 × B73) or paternal (B73 × OE1) parent. The dynamics of zein accumulation was analysed at three time points in the kernels produced by the F1 seed. The results show that the 15‐kDa β‐zein and 10‐kDa δ‐zein are strongly overproduced in kernels of either self‐pollinated OE1, or when OE1 was the maternal parent (Figure 8a), but not when OE1 was the paternal parent. To determine whether accumulation of these proteins correlates with an increased abundance of the respective mRNA, qRT‐PCR analysis was carried out on endosperm samples from developing kernels at 18 DAP. A range of zein mRNAs were measured. Figure 8b shows that the mRNAs corresponding to the 10‐kDa, 15‐kDa and 18‐kDa zeins were significantly more abundant in OE1. Interestingly, the 18‐kDa zein is another high Met + Cys zein. Although the mRNA for this protein was observed to increase when *AtSAT1* is maternally expressed (Figure 8b), this protein is not possible to detect in the gel shown in Figure 8a because it is obscured by the more abundant 19‐kDa α‐zein.

qRT‐PCR analysis of *AtSAT1* mRNA expression in embryo and endosperm from 18‐DAP kernels showed that *AtSAT1* accumulates mainly in leaf, but not in endosperm (Figure S4), as would be expected under transcriptional control of the *rbcS* promoter. We observed low‐level expression of *AtSAT1* in the seeds; therefore, the accumulation of these zeins is likely the function of *AtSAT1* expression in the vegetative tissues of the maternal plant.

### Maternal expression of *AtSAT1* overcomes the limitation in S‐amino acid supply needed for zein accumulation

**Figure 9:**
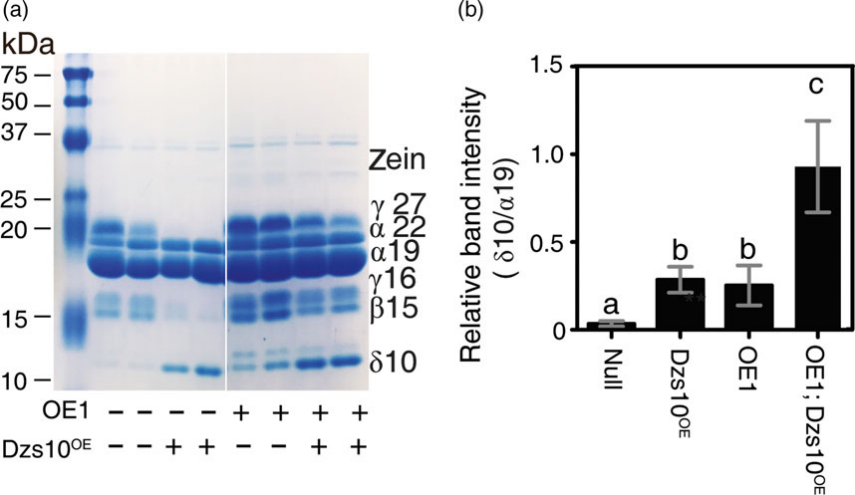
Zein profiles in hybrid kernels from a cross of OE1 with 10‐kDa δ‐zein overexpression maize line Dzs10^oe^. (a) Comparison of SDS‐PAGE zein profiles for Dzs10^oe^, OE1 and kernels from a OE1xDzs10^oe^ cross. The presence of the respective transgene is indicated under the gel. The zein profiles from two representative kernels are shown. (b) Band intensity of the 10‐kDa δ‐zein in the respective genotypes. The band intensity was measured by densitometry and normalized based on the amount of protein loaded on the gel. Each value is the average ±SD of measurements from six to eight kernels. Dzs10^oe^ is a transgenic maize line that overproduces the 10‐kDa δ‐zein, described in Lai and Messing (2002). The transgenic construct contains the Dzs10 coding sequence, but not the 5′ or 3′ UTRs. The UTRs are the targets of a post‐transcriptional regulation mechanism that controls the level of Dzs10 mRNA. Lacking the control targets 10‐kDa δ‐zein is overproduced (Lai and Messing, 2002).

One possibility for the pleiotropic effect of SAT overexpression on zein profile is that increased S‐amino acid supply influences accumulation of specific zein proteins in developing seed. Based on results from transgenic maize overexpressing the 10‐kDa δ‐zein (line Dzs10^OE^), it was earlier hypothesized that S‐amino acid supply limits the accumulation of high‐Met zeins (Lai and Messing, 2002; Wu *et al*., 2012). If so, then S‐amino acid supply limitation in Dzs10^OE^ should be overcome by *AtSAT1* expression. The results of this experiment are shown in Figure 9. A representative protein gel is shown in Figure 9a, and the densitometric quantification of the 10‐kDa δ‐zein is shown in Figure 9b. As Wu *et al*. (2012) previously reported, when the 10‐kDa δ‐zein is transgenically overexpressed in maize (Dzs10^OE^) the 10‐kDa δ‐zein accumulates, but the amounts of the 15‐kDa β‐ and 16‐kDa γ‐zeins are reduced (Figure 9a, compare null with Dzs10^OE^.). This observation was interpreted by Wu *et al*. (2012) to be a hallmark of S‐amino acid supply limitation. As we have reported here, *AtSAT1* expression results in increased 10‐kDa δ‐zein (compare null with OE1). The F1 progeny of the cross OE1 × Dzs10^OE^ produced seed in which expression of the 15‐kDa β‐ and 16‐kDa γ‐zeins are restored compared with the null. In addition, the 10‐kDa δ‐zein is increased further compared with the Dzs10^OE^ and OE1 transgenic parents (Figure 9b). This result strongly implies that S‐amino acid supply limits the accumulation of high‐Met zeins and that *AtSAT1* overcomes this limitation by increasing S‐amino acid supply.

## Discussion

In this study, we found that deregulation of S‐assimilation by ectopic overexpression of SAT in maize not only increases S‐assimilation (Figures 2, 3, 4 and S1, Table 1), but also increases the nutritional value of transgenic kernels (Figures 6 and 7). The increase in nutritional Met content resulted from a pleiotropic induction of expression of specific zeins (Figure 5) that are among those with the highest number of Met residues, including the 10‐kDa δ‐ and 15‐kDa β‐zeins. Maize also contains additional high Cys zein including the 50‐kDa, 27‐kDa and 16‐kDa γ‐zeins, but these were not increased to the same extent in SAT overexpressing lines (Figure 5). The high Met + Cys zeins usually make up only a small proportion of the total complement of seed storage proteins. It is for this reason that maize kernels are an insufficient source of Met necessary for a complete animal diet (Wu *et al*., 2012). As animals are able to metabolize Cys from Met, but are unable to perform the reverse reaction, Met is essential in diet but Cys is nonessential (Messing and Fisher, 1991).

The level of Met in kernels of *AtSAT1* line OE1 is as high as the inbred line BSSS53 that has been shown to contain sufficient Met to serve as a complete source for animal feed (Lai and Messing, 2002). The reason that other maize lines have lower levels of the 10‐kDa δ‐zein than BSSS53 is due to repression of the 10‐kDa δ‐zein mRNA by alleles of the transacting factor *dzr1* (Schickler *et al*., 1993). The alleles can exhibit either a dosage effect due to the triploid nature of the endosperm or are silenced due to imprinting (Chaudhuri and Messing, 1994). The imprinted allele can be activated, when maternally transmitted. Still in the hybrid seed business, repression by *dzr1* alleles in elite lines prevents the accumulation of sufficient levels of Met storage, making supplementation of feed with pure Met necessary, as has previously been shown (Messing and Fisher, 1991). Our results strongly support the hypothesis that S‐amino acid supply is limiting for expression of high‐Met zeins and influences the zein profile of maize kernels (Wu *et al*., 2012).

Prior work on overexpression of SAT in rice also noted an increase in protein‐bound Met content of grains (Nguyen *et al*., 2012). However, that study did not provide any indication of the regulation of seed storage proteins as we have found for high‐Met zeins in maize. Moreover, rice does not suffer from reduced nutritional value due to limiting Met content as maize does and rice also is not a source of animal feed like maize is (Rudra and Chowdhury, 1950).

When the 15‐kDa β‐zein was inserted into the potato genome, it increased both free Met content and the zein containing Met content of the transgenic tubers (Noctor *et al*., 1996). The current assumption is that β‐zein is post‐transcriptionally regulated by free Met (Bagga *et al*., 2005), although the mechanism is not understood, indicating that in other cases sulphur‐rich seed proteins need to be expressed in the appropriate background (Galili and Hofgen, 2002; Tabe and Droux, 2002). The present results show that S‐amino acid production in maternal parts of the plant can drive changes to the zein profile in the kernels of maize, indicating that S must be transported as a reduced organic form, and not in an oxidized inorganic form. This observation is supported by reciprocal cross‐experiments (Figure 8), and the observation that *AtSAT1*, engineered under transcriptional control of the *rbcS* promoter, is expressed in leaves, but only at very low level in developing kernels (Figure S4). Importantly, our results do not rule out that Cys synthesized in seeds may contribute to seed storage protein synthesis. Therefore, whereas sulphate is an abundant translocated S‐compound in plants (Buchner *et al*., 2010), the fraction that is transported to seeds cannot directly be the origin of the S that becomes incorporated into zeins in our experimental system. Rather, S‐amino acids synthesized in leaves must be translocated to the seed. Experiments using radioactive Met have indeed shown that Met exports from leaves in the form of *S*‐methyl‐L‐methionine (SMM) (Bourgis *et al*., 1999). SMM is a major S‐metabolite in the phloem of different plant species, and it is interconverted with Met in a pathway known as the SMM cycle via the enzyme methionine *S*‐methyltransferase (MMT) (Ranocha *et al*., 2001). However, more recently, it was shown that SMM cannot be a major transported S‐metabolite in maize because insertional *mmt* mutants grew normally, and seeds of the comparable *A. thaliana* mutant had normal S content (Kocsis *et al*., 2003). Therefore, the transport form of S must be some other reduced S‐compound. Another possibility is either Cys, cystine or both as has previously been shown in maize (Burgener *et al*., 1998), or glutathione as has been shown in rice (Kuzuhara *et al*., 2000). In this regard, grain crops may be different from legumes, where S‐amino acids for storage protein synthesis are likely synthesized *in situ* in seeds (Tabe *et al*., 2010). In total, our results support the model depicted in Figure S5, wherein sulphate, taken up by roots, is reduced and assimilated into Cys in leaf cells. Then, Cys, Met or some other derived metabolite(s) are transported to developing seeds where Cys and Met become incorporated into zeins during seed development.

In summary, this study shows that increasing S‐assimilation capacity in maize could be a critical approach for improving nutritional requirements for the enormous worldwide livestock and poultry feed market. The genetic engineering approach described here may provide a simple means to increase Met content in corn kernels in elite, commercial line of maize. Future optimization must be performed to determine the general applicability in commercial maize lines. Synthetic Met production for the formulation of livestock and poultry feed is expected to reach US$5.1 billion world market by 2024, so the technology provides a viable alternative for animal feed production. In addition, from a basic research standpoint, our results point to a regulation mechanism whereby the supply of S‐amino acids controls the production and accumulation of specific S‐sink proteins, the high Cys + Met zeins. This finding provides the impetus for mechanistic studies into the regulation mechanism.

## Experimental procedures

### Plasmid construction and plant transformation and initial analysis

The DNA primers used in this study are listed in Table S2. The *A. thaliana SAT1* cDNA (Gene Bank accession number BT008309.1) was used as template for PCR amplification with primers AtSAT1PF1 and AtSAT1PR1. This primer pair introduced two restriction enzyme sites, *BamH* I and *Sac* I. A DNA fragment including the *rbcS1* promoter plus the *rbcS1* 5′UTR was amplified from pPTN533 (Sattarzadeh *et al*., 2010) with primers RbcsPF and RbcsFR. The primers used for the *rbcS1* promoter PCR included the restriction sequences for *EcoR* I and *Bcl* I. Isocaudameric *Bcl* I and *BamH* I sites on the *rbcS1* promoter and *SAT1* fragments were ligated, and the expression cassette was cloned into the *EcoR* I and *Sac* I restriction sites of binary vector pTF102. The construct was vetted by sequencing, and a confirmed isolate was used to transform *Agrobacterium tumefaciens* strain EHA101.

Maize transformations using HiII Parent B × Parent A F_1_ immature embryos were carried out by *Agrobacterium*‐mediated transformation as described in Frame *et al*. (2002). The primary transgenic plantlets were verified by PCR amplification with the primer combination AtSAT1750PF and AtSAT1750PR using genomic DNA as template.

The plants used for transformation were grown in a greenhouse, and the immature embryos were harvested at 12 DAP when they were between 1.5 mm and 2.0 mm long. Embryo cultures were infected with EHA101 harbouring the plasmid, and T_0_ plants were selected on 3 mg/L BIALAPHOS (PhytoTechnology Laboratories, Lenexa KS; Catolog: B1730). After transfer of plantlets to soil, they were retested for the presence of the transformation construct using PCR with genomic DNA as template and the primers indicated above.

Six independent transgenic events were produced (T_1_). In a preliminary analysis, these T_1_ plants were tested for the presence of the SAT protein by immunoblotting (Nguyen *et al*. 2012) using an antibody raised against AtSAT1 (Murillo *et al*., 1995). SAT enzyme activity was measured as described by Harms *et al*. (2000). APR activity was measured as described by Martin *et al*. (2005).

To produce transgenic lines for experimentation T_1_ transgenic plants were backcrossed to B73, and progeny identified showed 1 : 1 segregation for the transformation construct. Event number 1 (OE1) and 3 (OE3) were selected for detailed analysis. Both OE1 and OE3 were backcrossed with B73 for two generations then self‐fertilized to produce nonsegregating lines.

### Quantitative RT‐PCR

Quantitative RT‐PCR was carried out with the primers designated for this purpose in Table S2. Total RNA was extracted using TRIzol reagent (Invitrogen Inc., Waltham, MA) and purified with the RNeasy Mini Kit after DNase 1 digestion (Qiagen Inc., Germantown, MD). Quantitative RT‐PCR was performed in a BIO‐RAD CFX connect Real‐Time system using SYBR○R Premix Ex TaqTM (Tli RnaseH Plus) (Zhang *et al*., 2015). The maize *Actin* gene was used as a reference control, and the expression data were calculated using the method described by Livak and Schittgen (Livak and Schmittgen, 2001). Statistically significant differences in expression levels of genes were calculated using the Student's *t*‐test.

### Analytical methods

Analytical measurements were performed both on leaf and kernel samples. Individual leaf samples consisted of pools of five equal‐sized leaf discs taken from equidistant locations along the length of the blade of the youngest fully expanded leaf from 2‐month‐old plants. The samples were pulverized to a fine powder in liquid nitrogen and stored at −70 °C until analysis. Unless noted otherwise, individual kernel samples were mature dry seeds prepared from single maize ears as described by Wu *et al*. (2012). Zein and nonzein protein fractions were also prepared as described by Wu *et al*. (2012) using 70% (w/v) ethanol and 2% (w/v) 2‐mercaptoethanol. Nonzein proteins are in the alcohol‐insoluble fraction, and zein proteins are in the alcohol‐soluble fraction.

Total glutathione (reduced and oxidized) in leaf tissue was measured using the GSH‐Glo^TM^ Glutathione Assay Kit (Promega Inc., Madison WI; Catalog #V6911) using a method established for plant tissues as described in Zhang *et al*. (2014). Sulphite content of leaves was assayed spectrophotometrically with Grant's fuchsine reagent as described in Leinweber and Monty (1987). OAS content of leaves was measured using HPLC‐MS/MS (Applied Biosystems API 3200 QTRAP LC/MS/MSn mass spectrometer Ultimate3000 ‐ API 3200 Q‐TRAP, Haidian District, Beijing, China) by the Beijing Mass Spectrometry Medical Research Co. Ltd. (Beijing, China). Leaf material was extracted in H_2_O and then mixed with 4 volumes of methanol. After centrifugation at 16.1 rcf for 5 min, the supernatant was used for OAS measurement. Standard OAS (Cat no.: CDS020792‐25MG) was purchased from Sigma.

Soluble and total amino acid content of leaf and kernel samples was analysed by the Beijing Mass Spectrometry Medical Research Co. Ltd. Samples were pretreated with performic acid to yield the acid stable derivatives of Cys and Met, cysteic acid and methionine sulphoxide. Samples were acid hydrolysed to yield total amino acid content.

Biosensor measurement of kernel Met was carried out with a fluorimeter (Synergy 2, Bio‐Tek Instruments, http://www.biotek.com/) as described (Bertels *et al*., 2012), with the following modifications. Zein and nonzein protein fractions were adjusted to 300 μg/mL with water. In a 0.8 mL reaction, 0.1 mL of protein sample was combined with a protease mixture consisting of 22 m Units Type XIV protease (part number P5147, Sigma‐Aldrich, St. Louis, MO) and Aminopeptidase M (part number 164598, Sigma‐Aldrich) in 10 mm sodium phosphate buffer pH 7.0. The reaction was incubated at 37 °C for 2 h. M9 minimal medium was used in place of MMAB medium. Met content is presented as per cent amino acid (%AA) calculated by dividing moles of Met by moles of amino acids in the protein sample, assuming an average amino acid molecular weight of 110.

### Chicken feeding experiment

Mature dry maize seeds were harvested from field‐grown plants (13.6 Kg of B73 and 12 Kg of OE1) and ground into powder. The samples were used as the cornmeal source in a feeding trials carried out at Sichuan Agricultural University (Chengdu, China).

The cornmeal was incorporated into a feed that was formulated as follows: each 100 g of feed contained 55.4 g corn meal, 36.2 g of soya bean meal, 0.08 g of L‐Lys, 0.25 g of NaCl, 3.9 g of corn oil, 1.0 g of vitamin mixture (Guangdong Dahuanong Animal Health Products Co., Ltd., Xincheng, China), 80 mg of choline, 1.2 g of calcium carbonate, 1.92 g of calcium hydrophosphate. Using commercial cornmeal, this diet has been shown to produce Met deficiency (Messing and Fisher, 1991). Newly hatched Erlang mountain chicks were randomly divided into five groups of four chicks per feed formulation and were fed with B73 corn formulation, or the formulation made from OE1. Feeding continued for 21 days, and the chicks were weighed at the start of the experiment and then at 7, 14 and 21 days. At 21 days, the chicks were photographed to document feather quality.

## Supporting information


**Figure S1** SAT enzyme activity, *AtSAT1* immunoblot, and zein profile of *AtSAT1* transgenic maize.

**Figure S2** Analysis of nonzein proteins in endosperm and embryo.

**Figure S3** Performance of transgenic line OE1 under field conditions.

**Figure S4** AtSAT1 expression pattern.

**Figure S5** Diagram of a flowering maize plant illustrating the relationship between SAT and zein accumulation.

**Table S1** Growth performance of chickens fed with corn meal from OE1.

**Table S2** Primers used in this study.
