# Supplementary figures and images for: Overexpression of serine acetyltransferase in maize leaves increases seed‐specific methionine‐rich zeins
Source: Plant Biotechnol J. 2017 Nov 29;16(5):1057–67. doi: 10.1111/pbi.12851 (PMC5902772; doi:10.1111/pbi.12851)

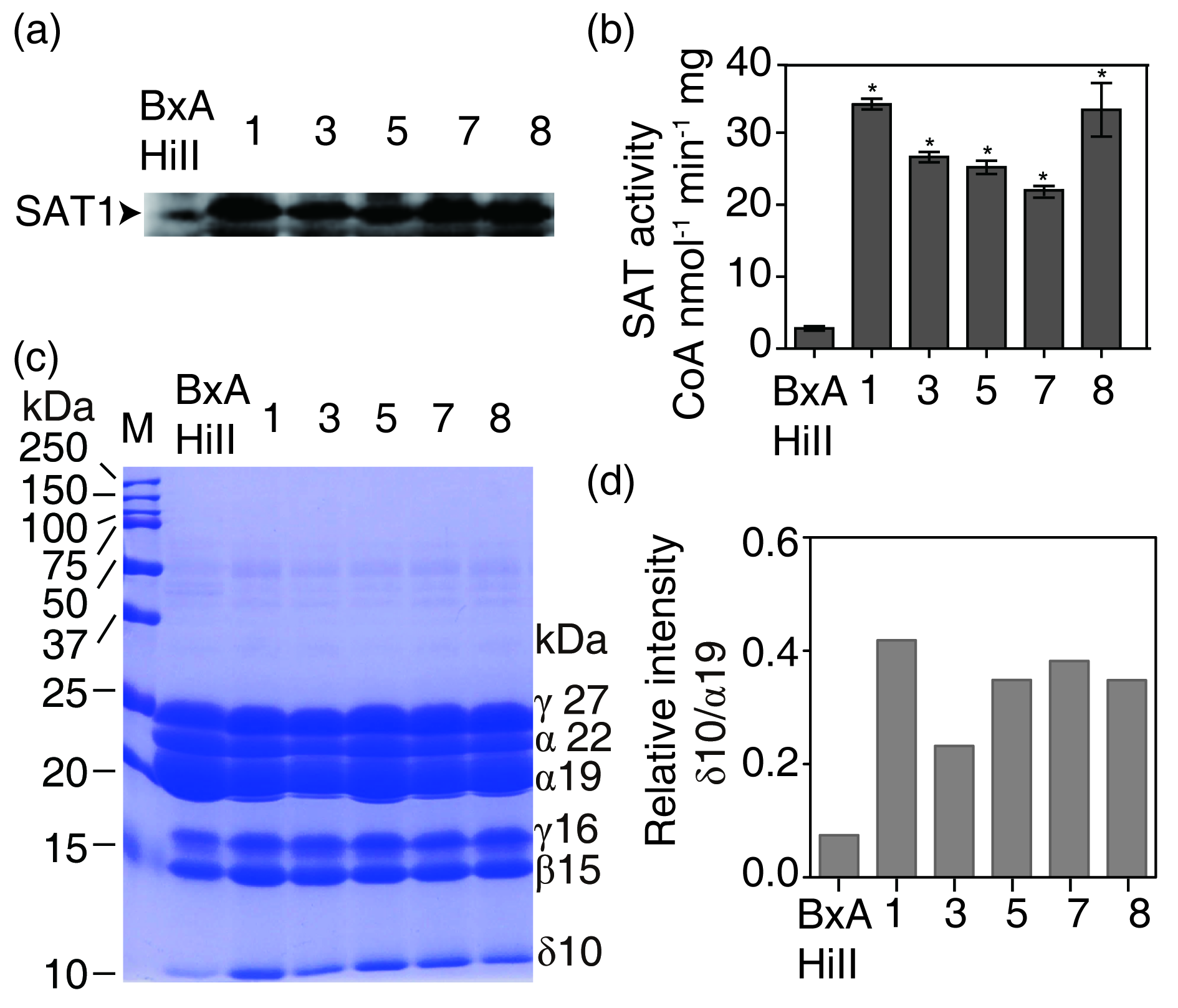

Supplement: Supplementary file 1 — Figure S1 SAT enzyme activity, AtSAT1 immunoblot, and zein profile of AtSAT1 transgenic maize. [file PBI-16-1057-s005.tif]

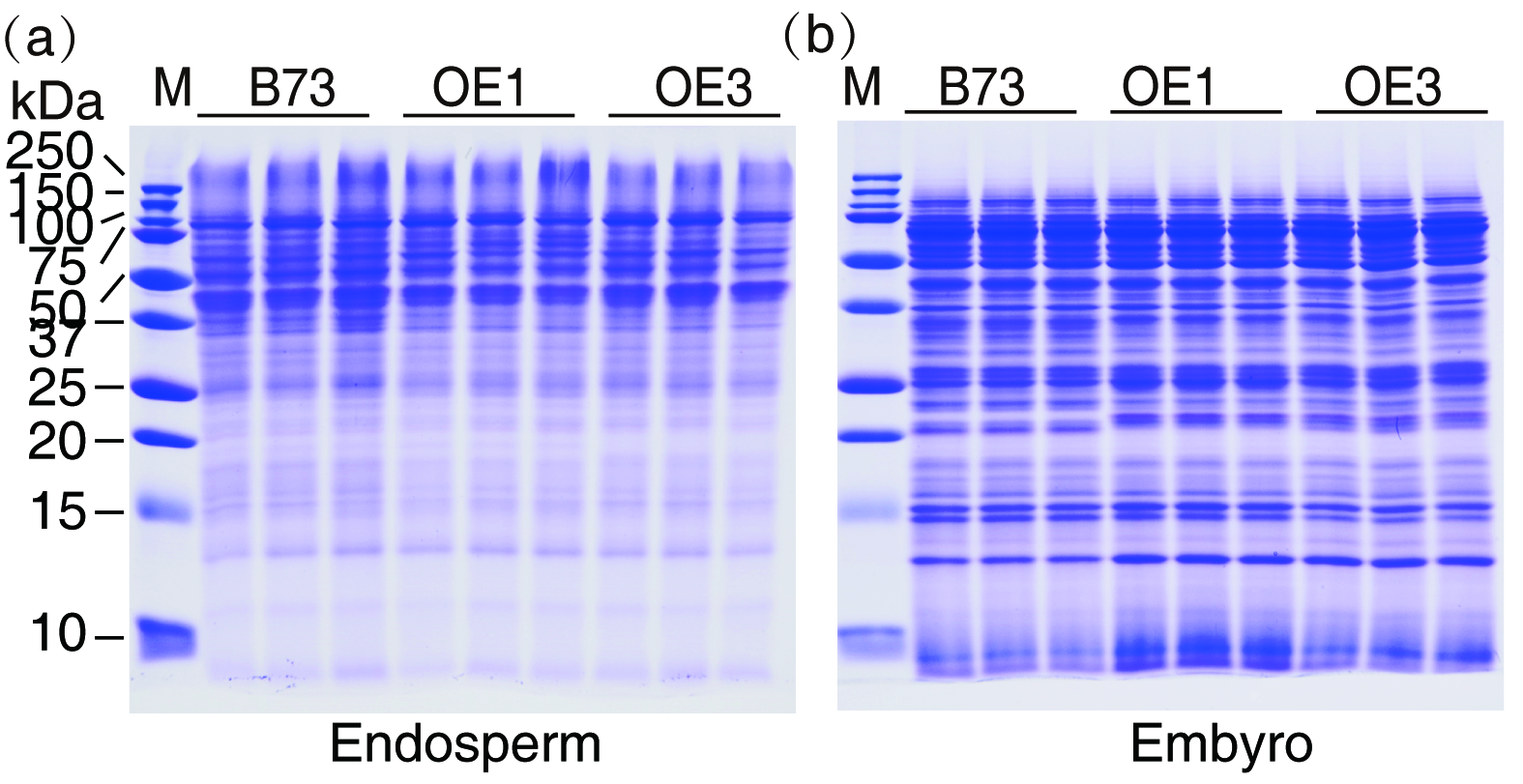

Supplement: Supplementary file 2 — Figure S2 Analysis of nonzein proteins in endosperm and embryo. [file PBI-16-1057-s007.tif]

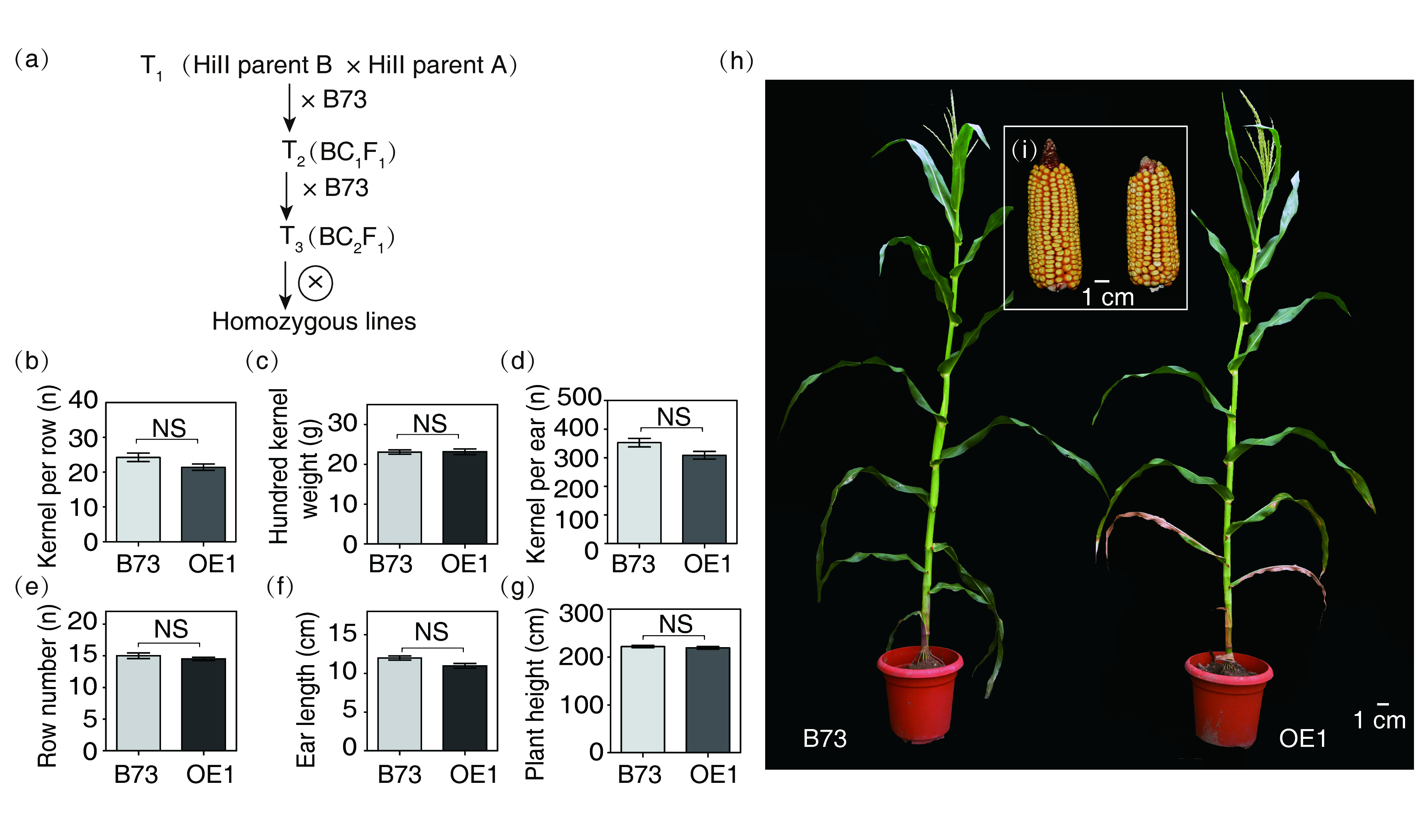

Supplement: Supplementary file 3 — Figure S3 Performance of transgenic line OE1 under field conditions. [file PBI-16-1057-s004.tif]

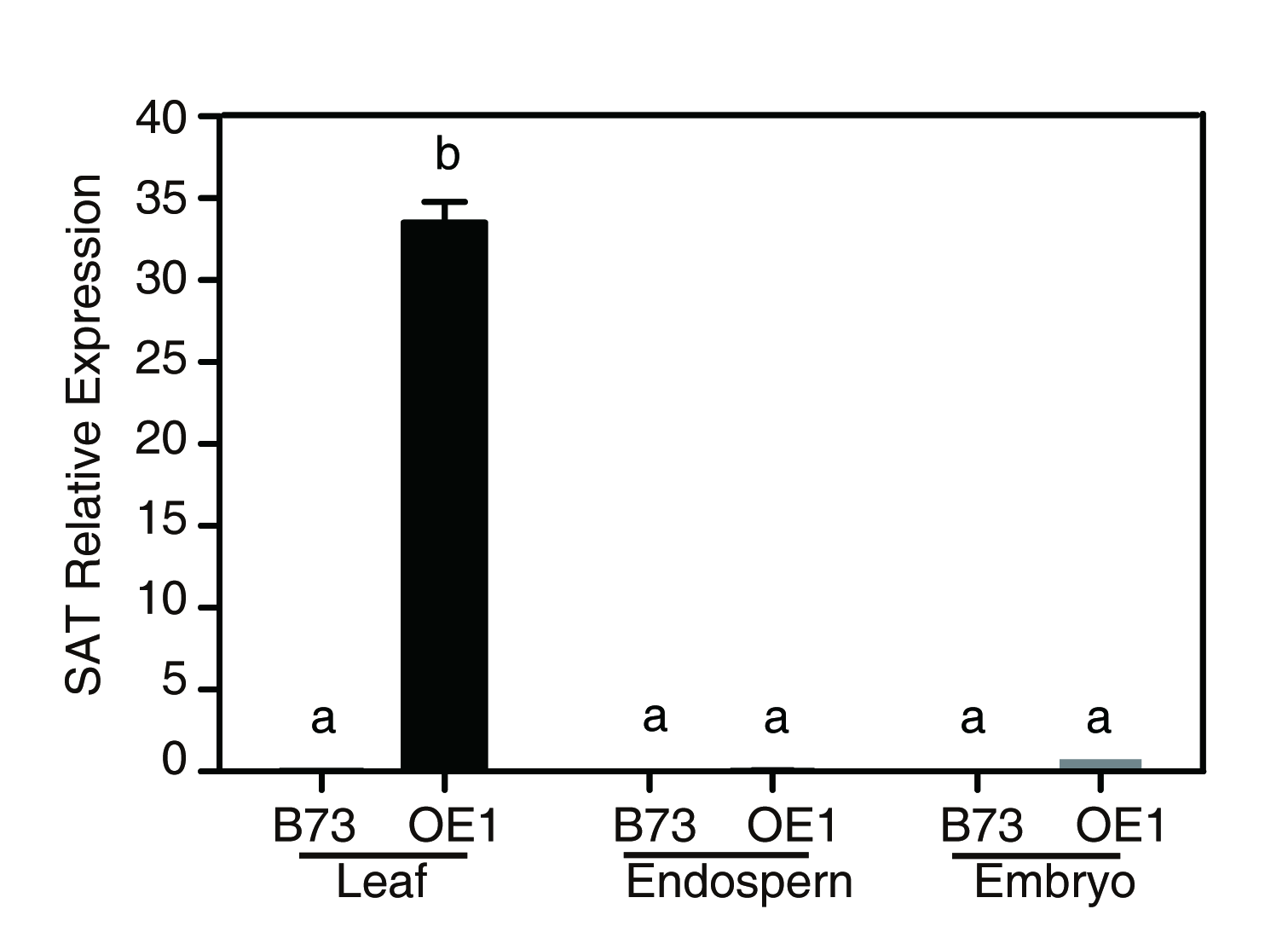

Supplement: Supplementary file 4 — Figure S4 AtSAT1 expression pattern. [file PBI-16-1057-s001.tif]

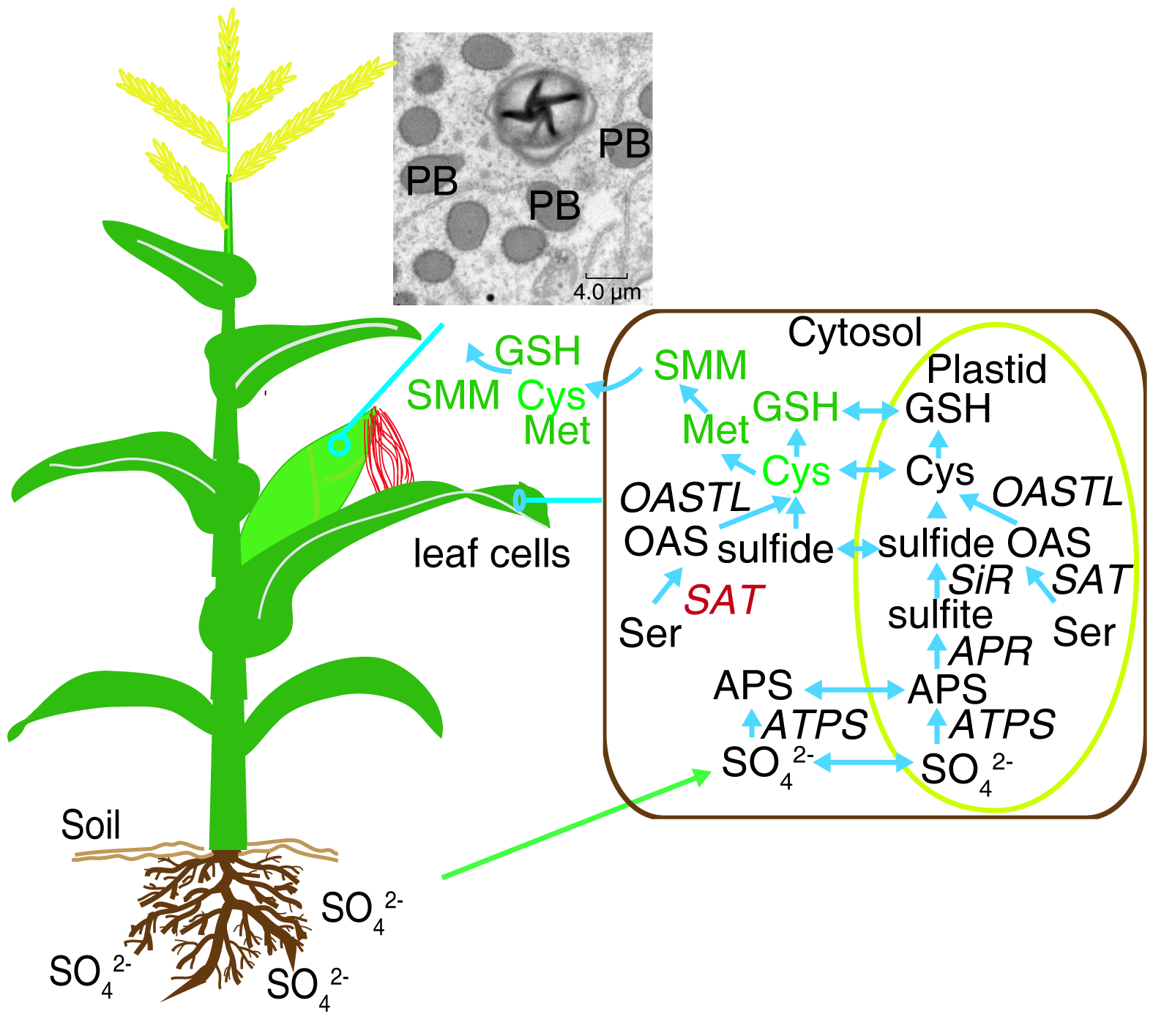

Supplement: Supplementary file 5 — Figure S5 Diagram of a flowering maize plant illustrating the relationship between SAT and zein accumulation. [file PBI-16-1057-s002.tif]
