# Supplementary material for: Overexpression of serine acetyltransferase in maize leaves increases seed‐specific methionine‐rich zeins
Source: Plant Biotechnol J. 2017 Nov 29;16(5):1057–67. doi: 10.1111/pbi.12851 (PMC5902772; doi:10.1111/pbi.12851)
Supplement: Supplementary file 6 — Table S1 Growth performance of chickens fed with corn meal from OE1. [file PBI-16-1057-s003.docx]

**Table S1. Growth performance of chickens fed with corn meal from OE1**

| Groups | Body weight (±SD) | | | | Weight increase | Food consumed | Efficiency |
| --- | --- | --- | --- | --- | --- | --- | --- |
|  | day 1 | day 7 | day 14 | day 21 |  |  |  |
| Normal | 42.42 (3.65)^A^ | 69.38 (5.51)^A^ | 116.14 (13.21)^A^ | 164.67 (28.50)^A^ | 144.46 (36.90)^A^ | 310.39^A^ | 0.46^A^ |
| Test | 43.38 (3.05)^A^ | 69.95 (6.40)^A^ | 143.71 (15.88)^B^ | 207.95 (30.17)^B^ | 164.57 (29.09)^B^ | 314.88^A^ | 0.52^A^ |

Feeding began with 1-d-old animals ranging in weight from 37 to 48 g, 20 animals per trial. Body weights were determined after the indicated days in 5 groups of 4 animals each. Food consumption in grams was also recorded. The values are the average weight per animal ±SD in parentheses. For each measurement date significant differences between trials (Student’s *t*-test, *p* < 0.01) are indicated with different letters (A,B). The weights that are not different are indicated with the same letter (A). Growth efficiency was calculated by dividing g weight gain by g food consumed.
