# Supplementary material for: Overexpression of serine acetyltransferase in maize leaves increases seed‐specific methionine‐rich zeins
Source: Plant Biotechnol J. 2017 Nov 29;16(5):1057–67. doi: 10.1111/pbi.12851 (PMC5902772; doi:10.1111/pbi.12851)
Supplement: Supplementary file 7 — Table S2 Primers used in this study. [file PBI-16-1057-s006.docx]

**Supplemental Table 2. Primers used in this study**

| Primer name | Sequences (5'to 3') | Note |
| --- | --- | --- |
| AtSAT1PF1 | GGATCCAACAATGGCAATCGAAGATGACGATGATGTCTGGAT | Cloning AtSAT1 |
| AtSAT1PR1 | GAGCTCTAAATCACATAATCAGACCACTCGG |  |
| RbcsPF | CAATTGGAGCTCCCTTTAATCTGGCGCTAGAT | Cloning Rbcs |
| RbcsFR | TGATCATAGTAGGCTCCCCCACACCGACGGCATAT |  |
| AtSAT1750PF | GCCACTCGTCCCACATCCGCT | Confirm AtSAT1 construct insertion in maize genome |
| AtSAT1750PR | CCTGTTCCTCCCAAGGTCACTCC |  |
| AtSAT1PF2 | CGAATCCACGAAGCAAGA | Quantitative RT-PCR of AtSAT1 gene expression |
| AtSAT1PF2 | TGTTTCCAGAGGGTATGAG |  |
| Actin PF | GCTACGAGATGCCTGATGGTC | Reference gene for Quantitative RT-PCR |
| Actin PR | CCCCCACTGAGGACAACG |  |
| 27kDF1 | TGCCTACAGCCGTCTCG | Quantitative RT-PCR of 27-kDa zein gene expression |
| 27kDR1 | GAGGGCAACGAGCAACAC |  |
| 22kDF1 | TTCCACAATGCTCACTTGCT | Quantitative RT-PCR of 27-kDa zein gene expression |
| 22kDR1 | GTTGTTGTAAGACGCTCGCC |  |
| 19kD-AF1 | GCTCCTTGGTCTTTCTGCAA | Quantitative RT-PCR of 19 kDa zein gene three copies expression |
| 19kD-AR1 | GGTAACTGCTGTAATAGGGCTGATG |  |
| 19kD-BF1 | CCAGCCCTATCTTTGGTGCA |  |
| 19kD-BR1 | TCAGTGCGGCCAATTGGTTA |  |
| 19kD-DF1 | GCACAACAACTACAACAACA |  |
| 19kD-DR1 | AATGGTAGTAGCTGTTGTGC |  |
| 18kDaF | TGCCTTCGACGATGACGCC | Quantitative RT-PCR of 18-kDa zein gene expression |
| 18kDaR | GATGGAATCAGAGTAAC |  |
| 16kDaF | CGGCGGTGTCTACTACTGAG | Quantitative RT-PCR of 16-kDa zein gene expression |
| 16kDaR | GGTTCATTCAGGTCATTGCTC |  |
| 15kDaF1 | CTACCGCACCAACCCCTG | Quantitative RT-PCR of 15-kDa zein gene expression |
| 15kDaR1 | TCAAGCGGCCGATAGATTC |  |
| 10kDaF | AGATGATGACGCCTAACA | Quantitative RT-PCR of 10-kDa zein gene expression |
| 10kDaR | ATGAATGGTAACTGCTG |  |
| P27pro:Zps10_for | 5’-CATCGACGTGCTACGTAAAGAGAG-3’ | Confirmation of Dz10^oe^ transgenic plants |
| P27pro:Zps10_rev | 5’-ATCATGTTAGGCGTCATCATCTGT-3’ |  |
